# American Surgical Society

**Published:** 1879-06-20

**Authors:** 


### *AMERICAN SURGICAL SOCIETY.*

During the sitting of the American Medical Association, Prof. Gross of Philadelphia proposed the organization of an American Surgical Association, and a committee was appointed to report a constitution and by-laws. Dr. L. A. Dugas, of Georgia, is the Chairman, and Dr. Watson of New York, Secretary of the Committee.

